# A Squaramide-Based Organocatalyst as a Novel Versatile Chiral Solvating Agent for Carboxylic Acids

**DOI:** 10.3390/molecules29102389

**Published:** 2024-05-19

**Authors:** Fabio Spiaggia, Gloria Uccello Barretta, Anna Iuliano, Carlo Baldassari, Federica Aiello, Federica Balzano

**Affiliations:** 1 Department of Chemistry and Industrial Chemistry, University of Pisa, Via G. Moruzzi 13, 56124 Pisa, Italy; anna.iuliano@unipi.it (A.I.); c.baldassari@studenti.unipi.it (C.B.); federica.balzano@unipi.it (F.B.); 2 National Research Council, Institute for Chemical and Physical Processes (CNR-IPCF), Via G. Moruzzi 1, 56124 Pisa, Italy; federica.aiello@cnr.it

**Keywords:** squaramide, chiral analysis, chiral auxiliary, NMR, enantiodiscrimination, diffusion coefficient, Overhauser effect

## Abstract

A squaramide-based organocatalyst for asymmetric Michael reactions has been tested as a chiral solvating agent (CSA) for 26 carboxylic acids and camphorsulfonic acid, encompassing amino acid derivatives, mandelic acid, as well as some of its analogs, propionic acids like profens (ketoprofen and ibuprofen), butanoic acids and others. In many cases remarkably high enantiodifferentiations at ^1^H, ^13^C and ^19^F nuclei were observed. The interaction likely involves a proton transfer from the acidic substrates to the tertiary amine sites of the organocatalyst, thus allowing for pre-solubilization of the organocatalyst (when a chloroform solution of the substrate is employed) or the simultaneous solubilization of both the catalyst and the substrate. DOSY experiments were employed to evaluate whether the catalyst–substrate ionic adduct was a tight one or not. ROESY experiments were employed to investigate the role of the squaramide unit in the adduct formation. A mechanism of interaction was proposed in accordance with the literature data.

## 1. Introduction

The pursuit of new, efficient and versatile direct methods for enantiomer differentiation remains a central focus of chemical researchers, with both enantioselective chromatography [1,2,3] and nuclear magnetic resonance (NMR) spectroscopy [4,5,6,7,8] playing a preeminent role. NMR-based methods for chiral analysis rely on the use of chiral auxiliaries devoted to remove inherent isochrony of enantiomers by converting them into intrinsically anisochronous diastereomers. This is achieved through the use of chiral derivatizing agents (CDAs) to produce covalently bonded diastereomeric derivatives, and chiral solvating agents (CSAs) or chiral lanthanide shift reagents (CLSRs), diamagnetic and paramagnetic, respectively, to differentiate enantiomers via noncovalent interactions occurring in solution. CSAs emerge for practicality of use since, like CLSRs, they are simply mixed with the chiral substrate in a suitable solvent directly in the NMR tube, and no significant line broadening effects are observed in the corresponding NMR spectra, in contrast to what typically observed with paramagnetic systems. The literature on CSAs is huge, spanning from low-molecular weight systems to flexible structurally complex molecules able to embrace differently sized chiral substrates, in favor of enantiodiscriminating versatility. In addition, preorganized rigid CSAs, being highly responsive to stereoelectronic features of stereoisomeric species, are endowed with enhanced enantiodiscriminating selectivity and efficiency. Within the realm of chiral auxiliaries harnessing noncovalent interactions in chiral discrimination processes, a foremost position is undoubtedly held by chiral liquid crystals, distinguished for their versatility [9,10]. Among low-molecular weight CSAs, amides represent historically [6,11] preeminent CSAs by virtue of their hydrogen bond donor-acceptor features. Subsequently, the development of CSAs with improved NH acidity and, consequently, enhanced hydrogen bond donor propensity was achieved as seen in ureas [12], and even more so in thioureas [13,14]. These functionalities have been embedded in several kinds of chiral platforms, both synthetic and coming from natural sources [15].

The remarkable significance of chiral carboxylic acids as constituents of numerous natural products and pharmaceutically relevant compounds has spurred the development of CSAs for NMR spectroscopy, primarily aimed at enantiodiscrimination within this compound class [4,5,6,7,16,17,18,19,20,21,22,23,24,25,26,27,28,29,30].

Squaramides are derivatives of squaric acid, in which the OH groups have been substituted with an NHR (secondary squaramide) or an NRR’ group (tertiary squaramide). They have proven to be applicable across various domains of chemical research, spanning from organocatalysis to supramolecular chemistry [31,32,33,34].

In particular, squaramide-based chiral compounds, which have emerged as efficient organocatalysts in several enantioselective reactions [31,33,34], are an unexplored CSA platform.

The squaramide scaffold has gained a lot of attention as a H-bonding donor–acceptor alternative to ureas and thioureas in organocatalysis [35]. Compared to the thiourea moiety, the squaramide is slightly larger, with a distance between the NH protons of around 2.7 Å; the structure of the cyclobutenedione ring also induces a convergent orientation of the NH groups by approximately 6 degrees [36]. Being endowed with two carbonyl groups, squaramides can act as a H-bond acceptor. Secondary squaramides, on the other hand, can also act as H-bond donors. It is recognized [37] that the donor/acceptor H-bond interactions between the squaramide unit and the substrate lead to a resonance-stabilized system [38,39]. Furthermore, the hydrogen bond donor features of the two NH groups, by virtue of their enhanced acidity, are superior to those of ureas and thioureas. These latter features (resonance stabilization and enhanced acidity) may lead to interaction energies far higher than those observed for ureas [37]. The hydrogen bonding donor capabilities of the squaramide units have been exploited for the synthesis of hydrogen bonding anion receptors to tackle malfunctioning ion channels, as first reported by Busschaert et al. [40]. Squaramide systems have also been used to coordinate carboxylate anions via hydrogen bonding, showing association constants toward the anions similar to those of urea systems [41].

**Figure 1:**
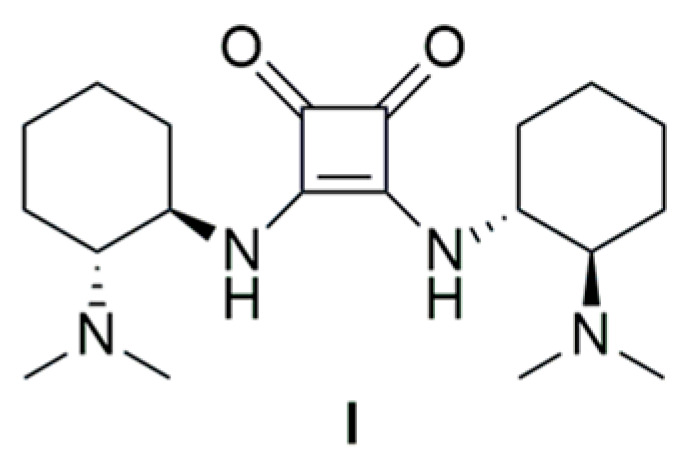
Chemical structure of organocatalyst **I**.

Kucherenko et al. prepared the bifunctional amine-squaramide system **I** represented in Figure 1 as an organocatalyst for enantioselective Michael reactions, together with other C2 symmetric squaramide–amine derivatives [42].

**Figure 2:**
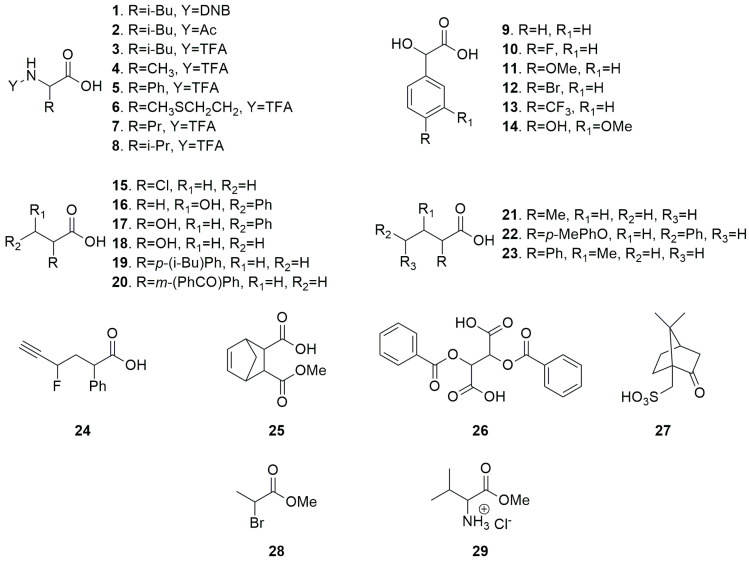
Structures of investigated substrates **1**–**29** (DNB = 3,5-dinitrobenzoyl, Ac = acetyl and TFA = trifluoroacetyl).

Although compound **I** is not the most efficient among the tested organocatalysts**,** it represents an attractive system owing to the presence of the squaramide unit and the two basic amine sites. Indeed, the electronic features of the squaramide unit make this moiety an interesting system, while the two basic amine sites can be protonated by acidic systems and work cooperatively with the two NH groups to form diastereomeric salts with chiral carboxylic acids. Such a kind of stereo-electronic features suggested to us to explore the potentialities of amine–squaramide **I** as a CSA for the NMR differentiation of polyfunctional carboxylic acids, including hydroxy acids and amino acids (Figure 2). In this paper, the NMR enantiodifferentiation of several carboxyl acids will be described. Nonequivalence (defined as ΔΔδ = |Δδ_R_ − Δδ_S_|, where Δδ_R_ and Δδ_S_ are the complexation shifts (δ_mixture_ − δ_free_) for the *R*- and *S*-enantiomer, respectively) data on different nuclei (^1^H, ^13^C and ^19^F) will be presented along with DOSY (diffusion-ordered spectroscopy) and ROESY (rotating-frame nuclear Overhauser enhancement spectroscopy) experiments that will serve as evidence of the interaction between compound **I** and the investigated substrates.

## 2. Results

### 2.1. Synthesis

**Scheme 1:**
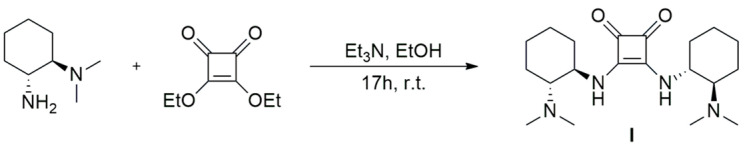
Synthesis of squaramide **I**.

Compound **I** was prepared according to Scheme 1, following the protocol of Kucherenko and co-workers [42], which was modified as far as reaction and work-up solvents are concerned.

The reaction, performed on a gram-scale, proceeded smoothly and the product, precipitated from the reaction mixture, was isolated into a chemically pure form by filtration. Starting from 0.575 g of 3,4-diethoxycyclobut-3-ene-1,2-dione (3.38 mmol) and using a slight excess of chiral diamine, 1.01 g of **I** (2.79 mmol, 82% yield) was obtained. Spectroscopic data are reported in the Materials and Methods section.

### 2.2. Enantiodiscrimination Experiments on Amino Acid Derivatives

Because of their importance in nature as chiral building blocks for proteins and peptides, the investigation of the enantiodiscriminating ability of compound **I** initially focused on amino acid derivatives as substrates. Deuterated chloroform (CDCl_3_) has been used as the main solvent for this investigation, as CDCl_3_ is a solvent of choice in NMR enantiodiscrimination experiments. Non-derivatized amino acids showed very low solubility even in the presence of squaramide **I** that, in principle, should favor solubilization by virtue of salification processes promoted by the basic *N*,*N*-dimethylamino moieties. Therefore, more soluble amino acid derivatives were examined in enantiodiscrimination experiments. Specifically, three kinds of *N*-derivatized amino acids were evaluated, containing a 3,5-dinitrobenzoyl (DNB, compound **1**), an acetyl (Ac, compound **2**) or a trifluoroacetyl (TFA, compounds **3**–**8**) moiety. Each one of them affords a probe group that generates simple (singlet for Ac and TFA) or well-recognized (high-frequency shifted triplet and doublet for DNB) resonances in the NMR spectra, facilitating the potential quantification of enantiomers. The TFA moiety also allows the detection of enantiomers in the ^19^F NMR spectra, where any interference at all is not produced by CSA signals.

Moreover, it is important to emphasize that compound **I** did show only slight solubility in CDCl_3_. In fact, the combination of a double H-bond acceptor and a double H-bond donor makes this class of compounds in general less soluble than the already low-soluble thioureas. Consequently, all enantiodiscrimination experiments were conducted either by solubilizing squaramide **I** with a solution of the substrate in CDCl_3_, or by adding the solvent to the CSA/substrate mixture. 

As compound **I** is endowed with two groups capable of capturing the substrate via H-bond/salification, the CSA/substrate stoichiometric ratios used for evaluating enantiomer differentiation were 1:1 and 1:2. Molar excesses of the CSA could not be explored due to its solubility issues.

**Figure 3:**
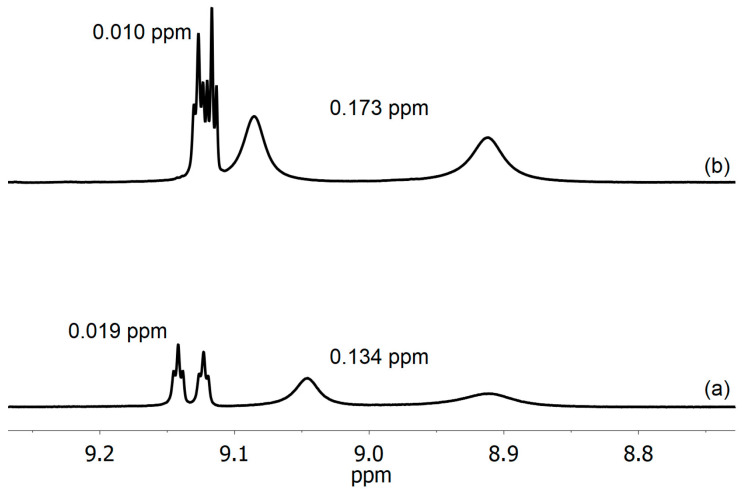
Spectral region of the ^1^H NMR spectra (600 MHz, CDCl_3_, 25 °C) including the resonances of the *para* and *ortho* protons of the racemic mixture of **1** in the presence of the CSA (10 mM) at the CSA/substrate stoichiometric ratios of: (**a**) 1:1 and (**b**) 1:2. Nonequivalence data are shown.

Firstly, three leucine derivatives, *N*-DNB (**1**), *N*-Ac (**2**) and *N*-TFA (**3**), were tested as potential substrates for squaramide **I**. The chiral auxiliary had rather singular effects on the resonances of the two enantiomers of DNB derivative **1**. Specifically, the *para* protons of the DNB group, which produced narrow triplets, exhibited a minor enantiomeric differentiation of 0.019 ppm, in comparison to a nonequivalence of 0.134 ppm measured for the broad resonances produced by *ortho* protons (Figure 3a). However, we found that at a 1:1 stoichiometric ratio, complete solubilization was not obtained. Therefore, the addition of an achiral tertiary amine was attempted as solubility promoter. DABCO was selected since it has been already employed as solubilizing agent in the presence of different classes of CSAs [14,25]. Complete solubilization was attained in the ternary equimolar mixture containing the CSA **I**, the substrate **1** and DABCO, but nonequivalences for *para* and *ortho* protons lowered to 0.014 ppm and 0.096 ppm, respectively. As an alternative to the use of a third solubility-promoting component, another equivalent amount of substrate was added to give a 2-to-1 molar ratio of **1**/CSA. In this manner, solubilization was complete and, once again, differentiation of the *para* protons lowered to 0.010 ppm. In contrast, enantiomeric differentiation of *ortho* protons increased to 0.173 ppm (Figure 3b), and the linewidth of the resonances of NH protons was so high that they became indistinguishable in the baseline. This behavior clearly indicates that the amide moiety is extensively involved in the interaction with squaramide, experiencing strong anisotropic effects likely originating from the conjugated squaramide systems.

In order to go more deeply into the origin of the aforementioned nonequivalences, we compared the diffusion coefficients obtained via DOSY experiments (see Materials and Methods) [43] for components in equimolar mixtures of **1**/CSA, both with and without DABCO (Table 1). It is noteworthy that, in the presence of CSA, the diffusion coefficient of substrate **1** remains quite unaffected by the presence of DABCO, suggesting a lower efficacy of the achiral base in comparison with the CSA in the salification process of the substrate. The diffusion coefficient of DABCO in the absence of other components is (16.3 ± 0.3) × 10^−10^ m^2^/s and decreases to (9.0 ± 0.2) × 10^−10^ m^2^/s in the presence of substrate **1**, signifying its capacity to bind to the substrate, salifying its carboxylic function. However, in the presence of the CSA in the ternary mixture **1**/CSA/DABCO, the diffusion coefficient of DABCO increases to (13.1 ± 0.2) × 10^−10^ m^2^/s. This suggests that the carboxylic group of substrate **1** is no longer totally available for salification by DABCO, as it is extensively engaged in the interaction with the dimethylamino basic groups of the CSA.

The decrease in nonequivalence in the presence of DABCO can be attributed to copresence of binding processes leading to a fraction of substrate **1** bound to DABCO. This somewhat compromises the substrate‘s ability to interact effectively with the CSA via salification. As a matter of fact, an effective interaction necessitates the cooperation between salification and H-bonding processes, involving both the dimethylamino groups and the enaminic portion of the squaramide. Therefore, the addition of DABCO is responsible for increasing the solubility of the substrate rather than intervening in the enantiodiscrimination process as observed with thioureas [14,25,26,27].

Organocatalyst **I** was also able to differentiate the enantiomers of the racemic mixture of substrate **2** (Table 2). Interestingly, at a 1:1 molar ratio the best differentiated protons of substrate **2** were the NH with a nonequivalence of 0.158 ppm and the methine proton bound to the stereogenic carbon (ΔΔδ = 0.116 ppm). The acetyl moiety was differentiated by 0.023 ppm. The addition of a further equivalent of substrate was detrimental since it led to a significant loss of differentiation (Table 2).

In Table 2, the enantioresolution quotient (E) was also reported. Indeed, as observed by Trujillo et al. [44], the parameter that better reflects the accuracy of the NMR quantitative determination of the enantiomeric composition is E rather than the nonequivalence. In fact, this parameter also takes into account the average linewidth of the signal (W) according to Equation (1):

E = |ΔΔδ|/(3W)
(1)

Indeed, minor nonequivalence measured for a simple singlet signal, such as that belonging to the acetyl protons, corresponds to an E that is significantly higher than 1.0.

**Figure 4:**
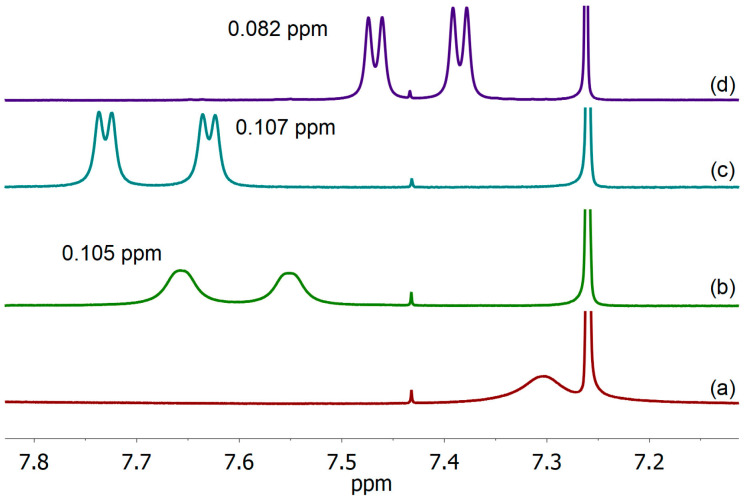
Spectral region of the ^1^H NMR (600 MHz, CDCl_3_, 25 °C) spectra including the NH resonance of substrate **3** in CSA/**3** mixtures ([CSA] = 10 mM) at different stoichiometric ratios: (**a**) 1:1; (**b**) 1:1.5; (**c**) 1:2; (**d**) 1:3. Nonequivalence data are shown.

Substrate **3** represented an attractive derivative, since *N*-TFA derivatives of amino acids show higher solubility in deuterated chloroform than their *N*-Ac and *N*-DNB counterparts. Figure 4 reports ^1^H NMR spectra of CSA/**3** mixtures at different stoichiometric ratios, along with nonequivalence data. The highest nonequivalence was obtained for the 1:2 and 1:1.5 CSA/substrate stoichiometric ratios (Figure 4b,c), but significantly more resolved signals are detected in the presence of two equivalents of substrate (Figure 4c). Further addition of substrate (CSA/substrate 1:3) led to a reduction of nonequivalence (Figure 4d).

**Figure 5:**
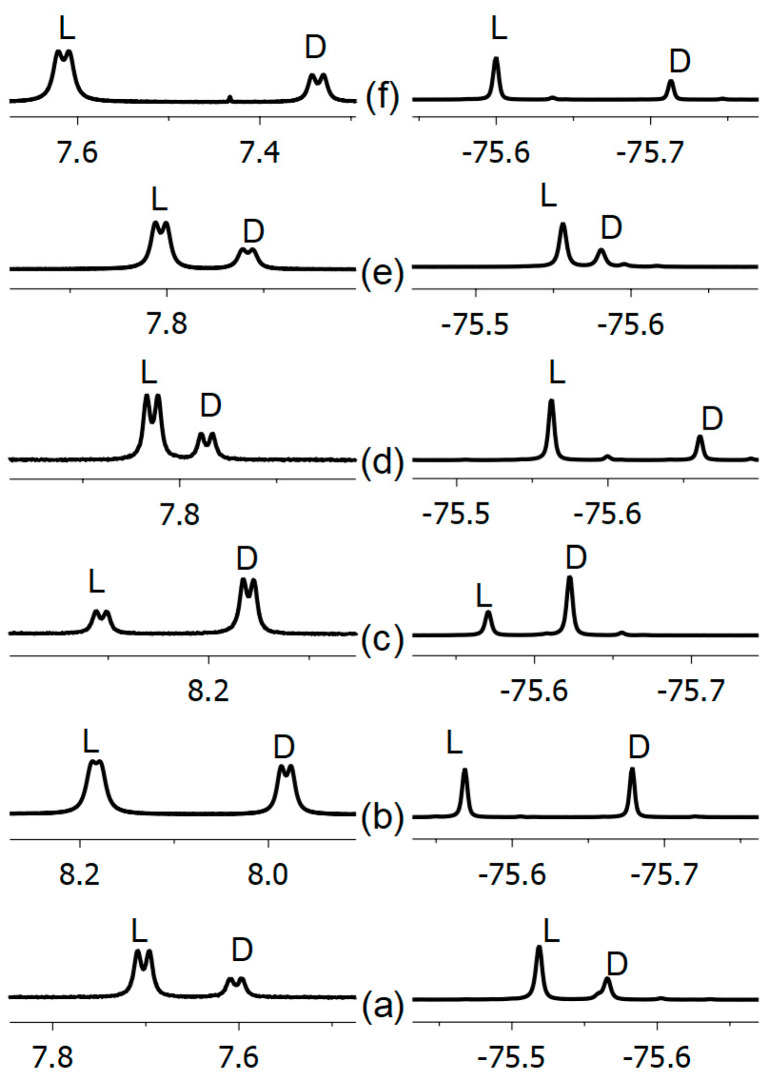
Spectral regions of (left) ^1^H NMR (600 MHz, CDCl_3_, 25 °C) and (right) ^19^F NMR (564 MHz, CDCl_3_, 25 °C) spectra involving amide and fluorine nuclei in 1:2 enantiomerically enriched mixtures of substrates **3**–**8**: (**a**) CSA/**3**; (**b**) CSA/**4**; (**c**) CSA/**5**; (**d**) CSA/**6**; (**e**) CSA/**7**; (**f**) CSA/**8**.

A valuable contribution to the study of enantiodiscrimination phenomena in the case of *N*-TFA derivatives stems from the presence of the trifluoroacetyl probe group, which can be observed in ^19^F NMR spectra, with well-resolved singlet signals. Therefore, the range of analysis for such types of derivatives was expanded by considering compounds **4**–**8** (Figure 2). Enantiodiscrimination experiments were carried out at the stoichiometric ratio of CSA/substrate 1:2 ([CSA] = 10 mM), which proved to be the best experimental conditions for measuring the highest nonequivalence in the case of substrate **3**. Table 3 summarizes the results obtained for substrates **3**–**8** and Figure 5 shows the corresponding ^1^H and ^19^F NMR spectral regions. 

Among the *N*-TFA amino acid derivatives analyzed, the best enantiodifferentiated substrate is the valine derivative, **8** (Table 3 and Figure 5). For all substrates, remarkably high nonequivalences were measured both for the amide proton (0.056–0.279 ppm) and ^19^F nuclei (0.025–0.113 ppm). Although the nonequivalences measured on NH protons are significantly higher than those measured on the ^19^F nuclei of CF_3_ groups, except for substrate **6**, the distinct spectral characteristics of the resonances of these two groups of nuclei must nevertheless be taken into account.

Due to the simpler signal structure of the fluorine nuclei (singlets) compared to the amide resonances (broad doublets), the calculated E factors (Table 3) highlighted how a better enantioresolution is achieved for ^19^F nuclei. For substrate **6**, with a better enantioseparation of fluorine signals compared to that measured on NH protons, the enantioresolution quotient for fluorine became approximately 8 times higher than that measured for the amide proton. In the case of the best enantiodifferentiated substrate **8**, for which the proton nonequivalence amply doubled the fluorine one, the value of E calculated for the amide signals was 2.3 times lower than that obtained for the fluorine nuclei.

**Figure 6:**
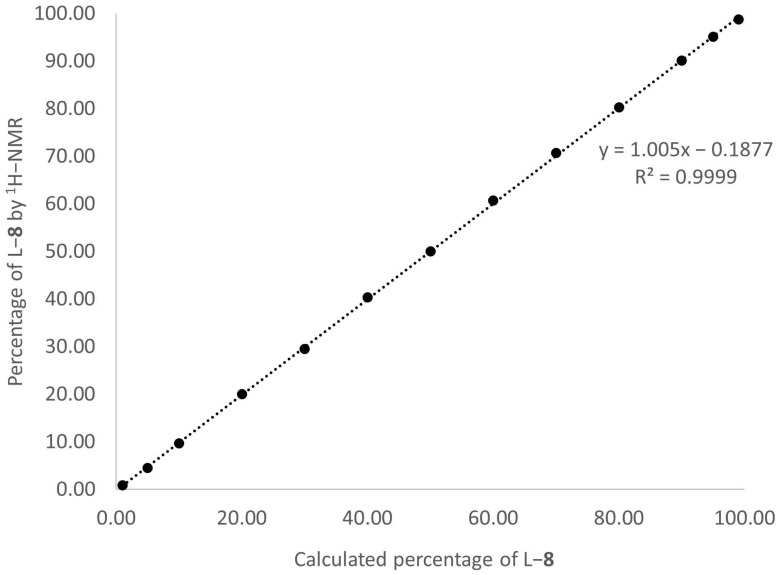
Correlation between the calculated percentage of l-**8** enantiomer against the one determined by integration via NMR.

When considering substrates **3**–**8**, the distinction between nonequivalence and enantioresolution quotient is not crucial. This is due to the fact that enantiomeric differentiations are notably high for both ^1^H and ^19^F nuclei. Additionally, the proton nuclei of the NH groups remain unaffected by interference from other signals produced by CSA. To verify the suitability of CSA for the quantitative determination of the enantiomeric composition, our focus was on the ^1^H NMR analysis of enantiomerically enriched mixtures via integration of the two NH signals produced by the enantiomers in the corresponding diastereomeric adducts. As an example, Figure 6 shows the good correlation (R^2^ = 0.9999) between the theoretical percentage of the l-**8** enantiomer and that determined via NMR. 

**Figure 7:**
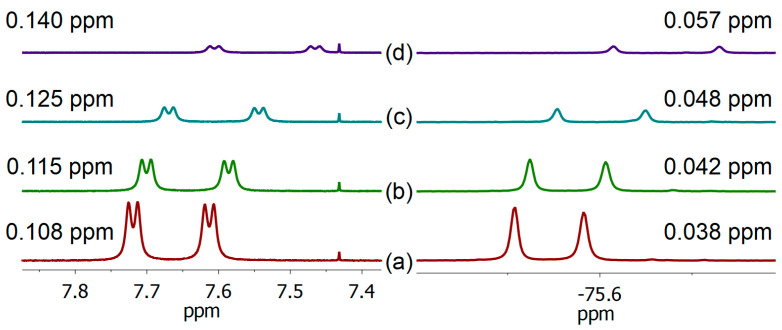
Spectral regions of (left) ^1^H NMR (600 MHz, CDCl_3_, 25 °C) and (right) ^19^F NMR (564 MHz, CDCl_3_, 25 °C) spectra involving amide and fluorine nuclei of substrate **3** in 1:2 mixtures of CSA/**3** with: (**a**) [CSA] = 10 mM; (**b**) [CSA] = 5 mM; (**c**) [CSA] = 2.5 mM; (**d**) [CSA] = 1.25 mM.

Based on the best CSA/substrate molar ratio experimentally found, the effect of the total concentration was also analyzed. Starting from a 1:2 CSA/**3** mixture ([CSA] = 10 mM), by diluting the solution and comparing the corresponding spectra (Figure 7), an increase of nonequivalence was observed. This trend was observed not only in the ^1^H NMR spectra, but also in the ^19^F NMR spectra (Figure 7). In all cases, baseline separation was detected. 

However, a different dependance of nonequivalence on total concentration was observed for the other substrates. For instance, the dilution of mixtures of CSA/**4** or CSA/**7** gave the same nonequivalence independent of the total concentration (Figures S2 and S3, Supplementary Materials). 

Another strength of CSA **I** is its capability to be utilized for configurational assignments of *N*-TFA amino acid derivatives. As a matter of fact, in enantiomerically enriched mixtures of substrates **3**–**8**, a reproducible correlation was found between the relative positions of enantiomeric signals and their absolute configuration. Specifically, for both the amide doublet and the fluorine singlet, the resonances at higher chemical shifts can be assigned to the l-enantiomer, while those at lower chemical shifts can be assigned to the d-enantiomer (Figure 5). Every *N*-TFA derivative behaved in this way (Figure 5), making compound **I** a reliable candidate for the configurational assignment of such compounds.

### 2.3. Enantiodiscrimination Experiments on Mandelic Acid and Its Analogs

Compound **I** was capable of discriminating the enantiomers of substrates **9** and several of its analogs (derivatives **10**–**14**, Figure 2). Preliminary experiments for compounds **9**–**11** at different stoichiometric ratios were aimed at finding the highest possible nonequivalence (Table 4 and Figures S4–S6, Supplementary Materials). As already stated for amino acid derivatives, substrate/CSA molar ratios greater than 1:1 were investigated since they guaranteed a complete solubilization of compound **I** in CDCl_3_. A 1:3 CSA/substrate ratio yielded the highest nonequivalence (Table 4) for all **9**–**11** substrates. Therefore, substrates **12**–**14** were analyzed directly at this CSA/substrate molar ratio. Nevertheless, the very low solubility of derivative **14** in deuterated chloroform, even in the presence of the CSA, allowed a maximum CSA/substrate molar ratio of 1:1.5 to be reached.

**Figure 8:**
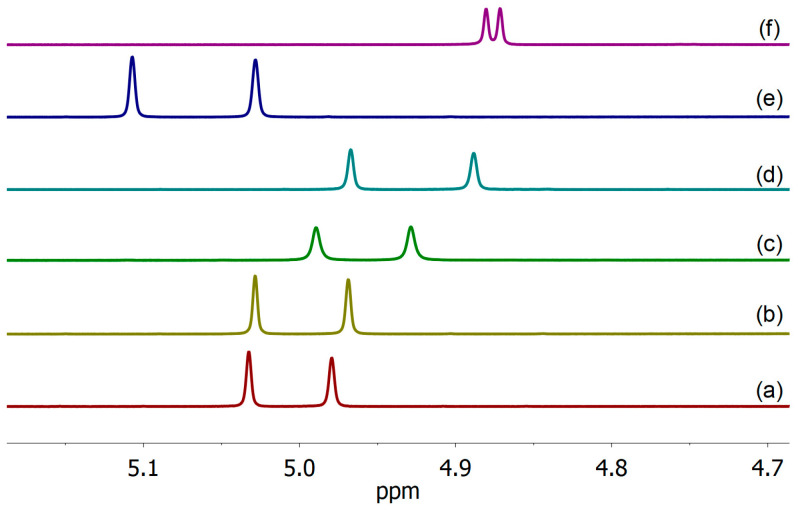
Spectral region of the ^1^H NMR (600 MHz, CDCl_3_, 25 °C) spectra, including the chiral methine proton of substrates **9–14** in 1:3 CSA/substrate mixtures ([CSA] = 5 mM): (**a**) **9**; (**b**) **10**; (**c**) **11**; (**d**) **12**; (**e**) **13**; (**f**) **14**.

Figure 8 shows the ^1^H NMR spectra of substrates **9**–**14** at the best CSA/substrate molar ratio (1:3).

For substrate **9**, the CH signal of one enantiomer broadened more than the other (Figure S7, Supplementary Materials). Carrying out a ^1^H-DOSY experiment led to the same value of the diffusion coefficient measured for the enantiomer resonating at a higher frequency as for that resonating at lower frequency ((7.0 ± 0.1) × 10^−10^ m^2^/s and (6.9 ± 0.1) × 10^−10^ m^2^/s, respectively). This demonstrates that the greater line broadening observed for the lower-frequency-shifted enantiomer was due not to a different binding strength to compound **I** but rather to different anisotropic effects at the CH protons caused by both the protonated dimethylamino groups and the squaramide unit of the CSA.

**Figure 9:**
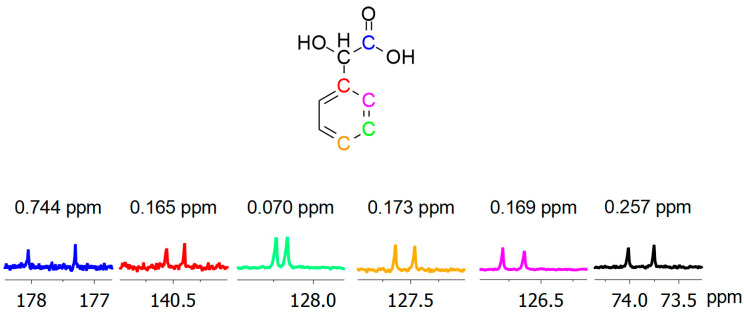
^13^C NMR (150 MHz, CDCl_3_, 25 °C) spectral regions of carbons of substrate **9** (15 mM) in the presence of the CSA (5 mM).

Moreover, ^13^C NMR analysis was explored for substrate **9** at the best CSA/substrate molar ratio (1:3). Outstanding nonequivalences, reported in Figure 9, were detected, with the aliphatic skeleton being the best enantiodifferentiated, to confirm the involvement of the polar moiety of the substrate in the H-bond/salification processes with the CSA.

**Figure 10:**
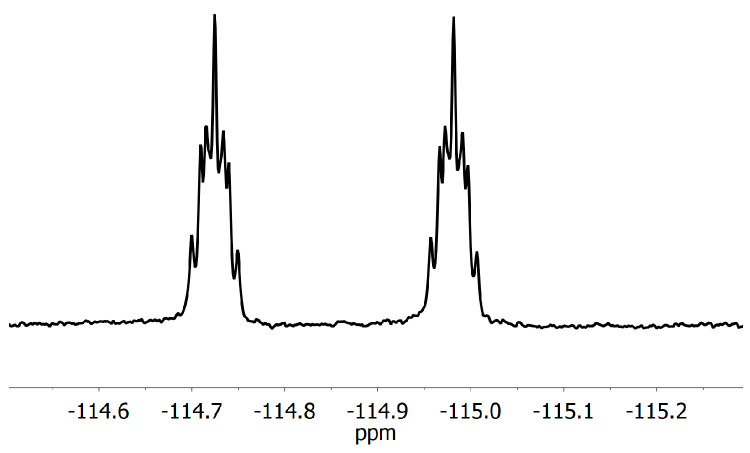
^19^F NMR (564 MHz, CDCl_3_, 25 °C) spectrum of substrate **10** (15 mM) in the presence of the CSA (5 mM).

For derivative **10**, a partial separation of the aromatic proton signals was also observed at the best molar ratio (CSA/substrate 1:3), together with an outstanding nonequivalence (0.258 ppm) for the ^19^F nuclei (Figure 10). 

### 2.4. Enantiodiscrimination Experiments on Other Acidic Systems

Compound **I** was also capable of differentiating the enantiomers of several propionic acids **15**–**20**, including profens like **19** and **20**, butanoic acids **21**–**23** and other acidic systems **24**–**27** (Figure 2).

Among propionic acids **15**–**20**, systems containing a hydroxy group yielded higher nonequivalences (Table S1, Supplementary Materials). In particular, substrates **16** and **17** were those providing baseline signal separation at the CSA/substrate stoichiometric ratio of 1:2 for at least one proton in the β-position with respect to the carboxylic function (chiral methine of substrate **16** and one of the two diastereotopic methylene protons of substrate **17**). The detected nonequivalences were 0.130 ppm (E = 1.7) and 0.076 ppm (E = 0.8) for substrates **16** and **17** (Figure S8 and Table S1, Supplementary Materials), respectively, which allowed a precise and accurate integration. Lactic acid (**18**) yielded controversial results due to the very low solubility of this acid, and also due to the presence of low-exchanging species as shown in Figure S9 (Supplementary Materials), where various signals were detected for methine and methyl protons in mixtures at different stoichiometric ratios. 

For ibuprofen (**19**) and ketoprofen (**20**), quite low enantioseparation was observed for aliphatic protons at all molar ratios analyzed (Table S1, Supplementary Materials). 

Among the investigated chiral butanoic acids, **21**–**23** (Figure 2), acid **22** with a 4-methylphenoxy moiety provided separation of some signals, with a baseline enantiodifferentiation of the methine proton of the chiral center (Table S2 and Figure S10, Supplementary Materials).

For substrate **24**, a C6 aliphatic carboxylic acid with a terminal alkynyl group, almost all protons were enantiodifferentiated with quite small nonequivalences independent of the CSA/substrate molar ratio (Table S2, Supplementary Materials).

**Figure 11:**
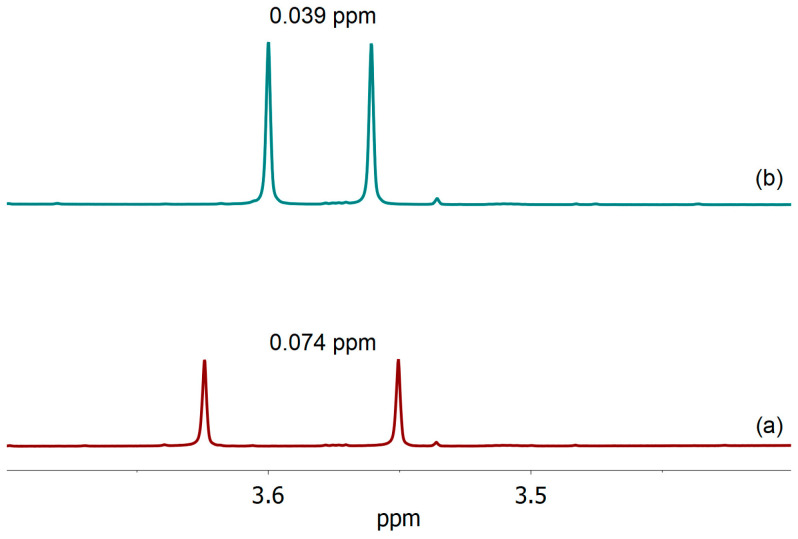
Methoxy spectral region of the ^1^H NMR (600 MHz, CDCl_3_, 25 °C) spectra of CSA (5 mM) and substrate **25** at the following CSA/**25** molar ratios: (**a**) 1:1; (**b**) 1:2.

CSA **I** was well able to differentiate chiral substrate **25**, as displayed in Figure 11.

Specifically, the 1:1 stoichiometric ratio provided an outstanding nonequivalence of 0.074 ppm (E = 13.4) for the methoxy protons. The 1:2 stoichiometric ratio caused the nonequivalence to decrease to 0.039 ppm (E = 6.7). 

**Figure 12:**
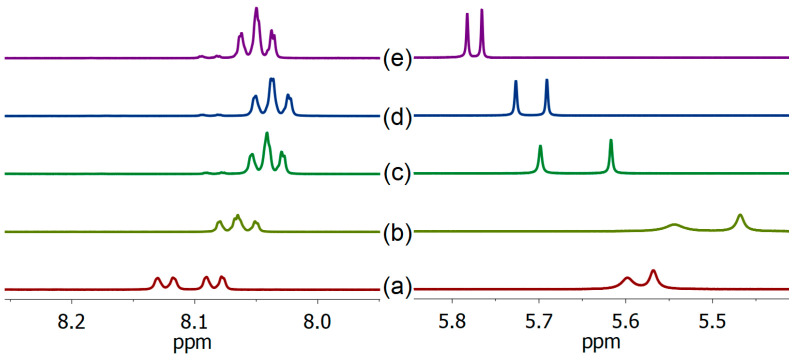
Spectral regions of the ^1^H (600 MHz, CDCl_3_, 25 °C) spectra including the aromatic *ortho* (left) and CH (right) signals of substrate **26** in CSA/**26** mixture at molar ratio of: (**a**) 2:1 ([CSA] = 10 mM); (**b**) 1:1 (([CSA] = 5 mM); (**c**) 1:2 ([CSA] = 5 mM); (**d**) 1:3 ([CSA] = 5 mM); (**e**) 1:4 ([CSA] = 5 mM).

Compound **I** was capable of providing enantiodifferentiation of substrates **26** and **27**. The tartaric acid derivative, **26** was endowed with two carboxylic groups and the stoichiometric ratio of 2:1 CSA/substrate was also investigated. Figure 12 shows that at this molar ratio, the *ortho* protons of substrate **26** (5 mM) were separated at the baseline. Increasing the relative content of the substrate led, however, to a reduction in the nonequivalence of the aromatic protons but to a marked increase in the separation of the CH signals up to the 1:2 CSA/substrate molar ratio (Figure 12c). 

Table 5 summarizes the results regarding the two resolving agents, **26** and **27**, investigated with squaramide **I** as the CSA.

### 2.5. Interaction Mechanism

The role of salification processes in the mechanism of interaction between CSA **I** and enantiomeric substrates has been fully confirmed through the analysis of mixtures containing substrates 2-bromopropionate (**28**) and valine methyl ester hydrochloride (**29**) (Figure 2). Both substrates **28** and **29**, devoid of free carboxylic functions, did not dissolve compound **I**. Hence, no discrimination of the two chiral substrates occurred in the presence of CSA **I**, thereby highlighting the significance of salification in this interaction mechanism.

1D-ROESY experiments (Figure S11, Supplementary Materials) were carried out on the 1:3 CSA/**9** mixture ([CSA] = 5 mM). The most notable, i.e., intermolecular, ROE effects were the ones between the CH proton of substrate **9** and the methyl protons of CSA **I**. Upon selective inversion of the *ortho* protons, an intermolecular ROE effect is observed with the methyl protons of compound **I**. Selective inversion of the methyl protons of squaramide **I** also shows a slight ROE enhancement on the *meta* protons of substrate **9**. 

**Figure 13:**
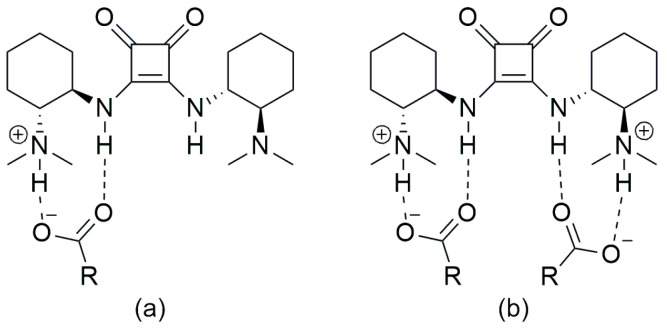
Possible interaction mechanism between compound **I** and a generic acid substrate for (**a**) 1:1 and (**b**) 1:2 stoichiometric ratios.

The interaction mechanism proposed herein is based on the well-recognized ability of the squaramide unit to work as a molecular receptor for anions [32,41] including carboxylate anions [45]. Essentially, it is plausible that the acid substrates protonate the two basic tertiary amine sites (hence, the necessity for stoichiometric ratios greater than 1:1 observed for the vast majority of the investigated substrates). The NH squaramide protons then act as a proton donor for the carboxylate anion in the way proposed in Figure 13.

## 3. Materials and Methods

### 3.1. Materials

Deuterated chloroform (CDCl_3_) used for the NMR experiments was purchased from Deutero GmbH (Kastellaun, Germany). All the following substrates were used without any further purification: substrates **1**, **15**, **18**–**21** and **26**–**27** (Sigma-Aldrich, Darmstadt, Germany); **2** (Thermo Scientific, Waltham, MA, USA); and substrates **9**–**14** and **16**–**17** (Alfa Aesar, Haverhill, MA, USA). Substrates **3**–**8** were synthesized according to reference [46]. Substrates **22**–**24** were available in our research laboratory. Substrate **25** was kindly provided by Prof. Alessandro Mandoli (University of Pisa, Italy).

### 3.2. NMR Methods

^1^H, ^19^F and ^13^C{^1^H} NMR measurements were carried out on a spectrometer operating at 600 MHz, 564 MHz, and 150 MHz for ^1^H, ^19^F and ^13^C nuclei, respectively. ^1^H and ^13^C chemical shifts are referred to tetramethylsilane (TMS) as the secondary reference standard, ^19^F chemical shifts are referred to trifluorotoluene as the external standard and the temperature was controlled (±0.1 °C). The proton spectra were recorded by using a π/2 pulse that was calibrated for each investigated system with a relaxation delay of 5 s. For all the 2D NMR spectra employed for the characterization of the CSA, the spectral width was the minimum required in both dimensions. The gCOSY (gradient correlation spectroscopy) map was recorded by using a relaxation delay of 1 s, 128 increments of 4 scans, each with 2000 data points. The 2D-ROESY (rotating-frame Overhauser enhancement spectroscopy) map was recorded by using a relaxation delay of 1 s, 128 increments of 16 scans, each with 2000 data points and a mixing time of 400 ms. The gHSQC (gradient heteronuclear single quantum coherence) and gHMBC (gradient heteronuclear multiple bond correlation) experiments were recorded with a relaxation delay of 1.2 s, 128 and 200 increments, respectively, with 8–16 scans, each of 2000 data points. The gHMBC experiments were optimized for a long-range coupling constant of 8 Hz. For the interaction mechanism study, 1D-ROESY spectra were recorded using a selective inversion pulse with scans ranging from 1024 to 6144, a relaxation delay of 1 s and a mixing time of 500 ms. DOSY (diffusion-ordered spectroscopy) experiments were carried out by using a stimulated echo sequence with self-compensating gradient schemes and with 64,000 data points. Typically, g was varied in 15 steps (4–256 scans) and Δ and δ were optimized to obtain an approximately 85–90% decrease in the resonance intensity at the largest gradient amplitude. The baselines of all arrayed spectra were corrected prior to processing the data. After data acquisition, each FID was apodized with a 1.0 Hz line broadening and Fourier-transformed. The data were processed with the DOSY macro (involving the determination of the resonance heights of all the signals above a pre-established threshold and the fitting of the decay curve for each resonance to a Gaussian function) to obtain pseudo two-dimensional spectra with NMR chemical shifts along one axis and calculated diffusion coefficients along the other. 

The synthesis of 3,4-bis(((1R,2R)-2-(dimethylamino)cyclohexyl)amino)cyclobut-3-ene-1,2-dione (**I**) was performed by slightly modifying the procedure reported in [42], to a solution of (1*R*,2*R*)-*N’*,*N’*-dimethylcyclohexane-1,2-diamine (1.01 g, 1.12 mL, 7.1 mmol) in ethanol (10.0 mL), triethylamine (1.9 mL, 13.5 mmol) and 3,4-diethoxycyclobut-3-ene-1,2-dione (0.575 g, 0.5 mL, 3.38 mmol) were added and the precipitation of a solid product was observed. The reaction mixture was stirred at room temperature for 17 h, then was filtered and the solid washed with ethanol (2 × 10mL) giving the pure product **I** as a solid (1.01 g, 2.79 mmol, 82% yield).

^1^H NMR (600 MHz, 40 mM, DMSO-d_6_ + 2 equiv of TFA, 25 °C), δ (ppm): 9.26 (br s, 2H, N*H*Me_2_^+^), 8.45 (br s, 2H, H3), 4.16 (br s, 2H, H4), 3.30 (br s, 2H, H5), 2.80 (br s, 6H, Me), 2.69 (br s, 6H, Me), 2.07 (d, ^2^J_8-’’_ = 11.6 Hz, 2H, H6), 1.99 (d, ^2^J_11-11′_ = 13.1 Hz, 2H, H9), 1.78 (d, ^2^J_10-1′’_ = 10.2 Hz, 2H, H8), 1.68 (d, ^2^J_9-9′_ = 10.2 Hz, 2H, H7), 1.49 (m, 2H, H6′), 1.43 (m, 2H, H9′), 1.31 (m, 2H, H8′), 1.27 (m, 2H, H7′). ^13^C NMR (150 MHz, 40 mM, DMSO-d_6_ + 2 equiv of TFA, 25 °C), δ (ppm): 182.3 (C1), 168.4 (C2), 67.0 (C5), 52.1 (C4), 41.9 (Me), 36.6 (Me), 33.9 (C9), 23.6 (C7), 23.4 (C8), 22.3 (C6).

In Figures S12 and S13 (Supplementary Materials), the proton and carbon NMR spectra of squaramide **I**, together with the numbering scheme and the signal assignment, are reported.

## 4. Conclusions

This paper demonstrated that organocatalyst **I** is capable of differentiating the enantiomers of several chiral carboxylic acids via NMR spectroscopy and, therefore, marks an interesting example of a versatile CSA for carboxylic acids. The interaction mechanism likely involves an acid–base reaction, with the tertiary amine sites of compound **I** acting as the proton acceptors. Methyl 2-bromopropionate (**28**) and valine methyl ester hydrochloride (**29**) were not discriminated by addition of squaramide **I**. This is likely due to the fact that the former lacks any acid function, while the latter is not soluble in CDCl_3_ and therefore unable to solubilize compound **I** and form diastereomeric adducts. Two are the distinctive features of CSA **I** compared to others [25,26,27] proposed for the enantiodiscrimination of carboxylic acid derivatives. Firstly, it inherently contains the basic *N*,*N*-dimethylamino groups required to mediate the proton transfer from the enantiomeric substrate to CSA itself. Secondly, and most notably, its remarkable versatility sets it apart. *N*,*N*-dimethylamino groups can be protonated and act as attractive points for acidic substrates. The attraction may stem from both the rapidity in which the proton transfer reaction occurs and the electrostatic attraction between the two ionic products. The second structural feature is the squaramide unit that can coordinate the carboxylate anion and form a resonance-stabilized adduct. It is plausible to infer that both factors (i.e., the electrostatic interaction and the resonance-stabilized adduct) may contribute to inducing pronounced anisotropic effects on the enantiomers of chiral carboxylic acids. Such effects contribute to the high enantiomeric differentiation.

The versatility of organocatalyst **I** in differentiating enantiomers of chiral carboxylic acids suggests that the squaramide unit should be explored as a novel interacting moiety in the field of NMR chiral analysis. Indeed, coupling the high capacity of the squaramide unit of accepting/donating hydrogen bonds with aptly chosen chiral platforms may lead to the synthesis of an entirely new class of squaramide-based CSAs.

## Figures and Tables

**Table 1:** Diffusion coefficients (D, ×10^10^ m^2^/s) determined for DABCO (10 mM) and for its equimolar binary (DABCO/**1**) and ternary (DABCO/**1**/CSA) mixtures and for equimolar **1**/CSA (10 mM) mixture. Configurational assignment of compound **1** is reported in Figure S1 in Supplementary Materials.

Sample	D (×10^10^ m^2^/s)			
	D_DABCO_	D_d-1_	D_l-1_	D_CSA_
DABCO	16.3 ± 0.3			
DABCO/**1**	9.0 ± 0.2	7.0 ± 0.1	7.0 ± 0.1	
DABCO/**1**/CSA	13.1 ± 0.2	5.8 ± 0.1	6.6 ± 0.1	6.4 ± 0.1
**1**/CSA		5.4 ± 0.1	5.8 ± 0.1	6.5 ± 0.1

**Table 2:** ^1^H NMR (600 MHz, CDCl_3_, 25 °C) nonequivalences (ΔΔδ, ppm) for the amide (NH), the chiral methine (CH) and the acetyl (Ac) protons of the racemic mixture of substrate **2** in the presence of the CSA (10 mM) in 1:1 and 1:2 CSA/**2** mixtures. Enantioresolution quotients are reported in parenthesis.

CSA/Substrate	ΔΔδ (ppm)		
	NH	CH	Ac
1:1	0.158 (1.6)	0.116 (1.0)	0.023 (3.5)
1:2	0.012 (0.1)	0.064 (0.5)	0.014 (2.1)

**Table 3:** ^1^H NMR (600 MHz, CDCl_3_, 25 °C) and ^19^F NMR (564 MHz, CDCl_3_, 25 °C) nonequivalences (ΔΔδ, ppm) for the amide (NH) and the trifluoromethyl (CF_3_) nuclei of substrates **3**–**8** (20 mM) in 1:2 CSA/substrate mixtures. Enantioresolution quotients are reported in parenthesis.

Substrate	ΔΔδ (ppm)	
	NH	CF_3_
**3**	0.100 (1.5)	0.047 (3.1)
**4**	0.201 (3.0)	0.110 (9.2)
**5**	0.147 (2.6)	0.052 (3.8)
**6**	0.056 (1.0)	0.098 (7.6)
**7**	0.091 (1.4)	0.025 (1.5)
**8**	0.279 (3.9)	0.113 (8.8)

**Table 4:** ^1^H NMR (600 MHz, CDCl_3_, 25 °C) nonequivalences (ΔΔδ, ppm) for the chiral methine proton of substrates **9**–**14** in the presence of CSA **I** (5 mM) at different molar ratios. Enantioresolution quotients are reported in parenthesis.

Substrate	CSA/Substrate	ΔΔδ (ppm)
**9**	1:2	0.044 (4.1)
	1:3	0.053 (4.8)
	1:4	0.047 (4.1)
**10**	1:2	0.057 (5.2)
	1:3	0.060; 0.258 * (5.6; 1.5 *)
	1:4	0.050 (4.9)
**11**	1:2	0.046 (3.1)
	1:3	0.061 (3.7)
	1:4	0.056 (4.5)
**12**	1.3	0.078 (6.0)
**13**	1:3	0.079; 0.021 * (6.2; 1.6 *)
**14**	1:1.5 **	0.009 (0.9)

* ^19^F (564 MHz, CDCl_3_, 25 °C) nonequivalence (ΔΔδ, ppm). ** Stoichiometric ratio determined via integration.

**Table 5:** ^1^H NMR (600 MHz, CDCl_3_, 25 °C) nonequivalences (ΔΔδ, ppm) for selected protons of racemic mixtures of substrates **26** and **27** in the presence of the CSA (5 mM) in several CSA/substrate mixtures. Enantioresolution quotients are reported in parenthesis.

Substrate	CSA/Substrate	ΔΔδ (ppm)			
		CH	Ar-CH*o*	Ar-CH*p*	Ar-CH*m*
**26**	1:1	0.076 (1.5)	0.016 (0.2)	0.004 (n.d.) *	0.008 (0.1)
	1:2	0.081 (7.2)	0.012 (0.2)	0.013 (0.1)	0.008 (0.1)
	1:3	0.036 (4.2)	0.014 (0.2)	0.010 (0.1)	0.003 (n.d.) *
	1:4	0.017 (2.4)	0.014 (0.2)	0.008 (0.1)	0.003 (n.d.) *
	2:1 **	0.030 (0.7)	0.040 (0.7)	0.020 (0.2)	0.025 (0.3)
**27**		CH_A_ ***	CH_B_ ***	CH_3_ (hf) ***	CH_3_ (lf) ***
	1:1	0.081 (1.0)	0.022 (0.3)	0.015 (1.6)	0.018 (1.9)
	1:2	0.009 (0.1)	0.020 (0.2)	0.004 (0.4)	0.007 (0.8)

* n.d. = not determined ** [CSA] = 10 mM. *** CH_A_ and CH_B_ are the two protons of the methylene bound to the sulfonic group; CH_3_ (hf) and CH_3_ (lf) are the methyl protons resonating at high and low frequency, respectively.

## Data Availability

Data are contained within the article or Supplementary Materials.

## Supplementary Materials

The following supporting information can be downloaded at: https://www.mdpi.com/article/10.3390/molecules29102389/s1, Figure S1: Spectral region of the ^1^H NMR (600 MHz, CDCl_3_, 25 °C) spectrum of compound **1** (10 mM) in equimolar CSA/**1**/DABCO mixture, including DNB resonances. Enantiomeric composition is 40% l- and 60% d-**1**; Figure S2: Spectral region of ^1^H NMR (600 MHz, CDCl_3_, 25 °C) spectra involving amide nuclei of substrate **4** in 1:2 mixtures of CSA/**4** with: (a) [CSA] = 10 mM; (b) [CSA] = 0.7 mM; Figure S3: Spectral region of ^1^H NMR (600 MHz, CDCl_3_, 25 °C) spectra involving amide nuclei of substrate **7** in 1:2 mixtures of CSA/**7** with: (a) [CSA] = 10 mM; (b) [CSA] = 5 mM; (c) [CSA] = 2.5 mM; (d) [CSA] = 1.25 mM; Figure S4: Spectral region of ^1^H NMR (600 MHz, CDCl_3_, 25 °C) spectra involving chiral methine of substrate **9** in mixtures of CSA/**9** ([CSA] = 5 mM) at the following CSA/substrate molar ratios: (a) 1:2; (b) 1:3; (c) 1:4; Figure S5: Spectral region of ^1^H NMR (600 MHz, CDCl_3_, 25 °C) spectra involving chiral methine of substrate **10** in mixtures of CSA/**10** ([CSA] = 5 mM) at the following CSA/substrate molar ratios: (a) 1:2; (b) 1:3; (c) 1:4; Figure S6: Spectral region of ^1^H NMR (600 MHz, CDCl_3_, 25 °C) spectra involving chiral methine of substrate **11** in mixtures of CSA/**11** ([CSA] = 5 mM) at the following CSA/substrate molar ratios: (a) 1:2; (b) 1:3; (c) 1:4; Figure S7: Spectral region of the ^1^H NMR (600 MHz, CDCl_3_, 25 °C) spectrum, including the chiral methine proton of substrate **9** in the presence of CSA (5 mM) at the stoichiometric ratio CSA/**9** 1:3.; Table S1: ^1^H NMR (600 MHz, CDCl_3_, 25 °C) nonequivalences (ΔΔδ, ppm) for selected resonances of racemic mixtures of substrates **15**–**20** in the presence of CSA **I** at different concentrations and molar ratios; Figure S8: Spectral region of the ^1^H NMR (600 MHz, CDCl_3_, 25 °C) spectra, including the chiral methine of substrate **16** (a) and one of the two diastereotopic methylene protons of substrate **17** (b) in the presence of the CSA (5 mM) at CSA/substrate stoichiometric ratio of 1:2; Figure S9: Spectral regions of ^1^H NMR (600 MHz, CDCl_3_, 25 °C) spectra, including the chiral methine (left) and methyl (right) protons of racemic mixtures of substrate **18** in the presence of the CSA (5 mM) at the following CSA/substrate stoichiometric ratios: (a) 1:1; (b) 1:2; (c) 1:3; (d) 1:4; Table S2: ^1^H NMR (600 MHz, CDCl_3_, 25 °C) nonequivalences (ΔΔδ, ppm) for selected resonances of racemic mixtures of substrates **21**–**24** in the presence of CSA **I** at different concentrations and molar ratios; Figure S10: Spectral region of the ^1^H NMR (600 MHz, CDCl_3_, 25 °C) spectrum including the chiral methine proton of substrate **22** in the presence of CSA (5 mM) at equimolar ratio; Figure S11: ^1^H NMR (600 MHz, CDCl_3_, 25 °C) spectrum of **9**/**I** mixture at the composition of 1:3 ([CSA] = 5 mM) (a) and 1D ROESY (600 MHz, CDCl_3_, 25 °C, mixing time = 500 ms) spectra of the same mixture corresponding to: (b) ortho-CH of substrate **9**; (c) CH of substrate **9**; (d) CH_3_ of squaramide **I**. Protons involved in intermolecular ROE effects are indicated in the spectra; Figure S12: ^1^H NMR (600 MHz, 40 mM, DMSO-d_6_ + 2 equiv of TFA, 25 °C) spectrum of compound **I**; Figure S13: ^13^C NMR (150 MHz, 40 mM, DMSO-d_6_ + 2 equiv of TFA, 25 °C) spectrum of compound **I**. * Indicate resonances belonging to TFA.
