# Supplementary material for: A Squaramide-Based Organocatalyst as a Novel Versatile Chiral Solvating Agent for Carboxylic Acids
Source: Molecules. 2024 May 19;29(10):2389. doi: 10.3390/molecules29102389 (PMC11123912; doi:10.3390/molecules29102389)
Supplement: Supplementary file 1 [file molecules-29-02389-s001.zip › molecules-2993429-supplementary.pdf]

A squaramide-based organocatalyst as a novel versatile chiral solvating agent for carboxylic acids

Fabio Spiaggia [1,*], Gloria Uccello Barretta [1,*], Anna Iuliano [1], Carlo Baldassari [1], Federica Aiello [2], and Federica Balzano [1]

[1] Department of Chemistry and Industrial Chemistry, University of Pisa, Via G. Moruzzi 13, 56124 Pisa, Italy; anna.iuliano@unipi.it (A.I.); c.baldassari@studenti.unipi.it (C.B.); federica.balzano@unipi.it (F.B.)

[2] National Research Council, Institute for Chemical and Physical Processes (CNR-IPCF), Via G. Moruzzi 1, 56124 Pisa, Italy; federica.aiello@cnr.it

* correspondence: f.spiaggia@studenti.unipi.it (F.S.); gloria.uccello.barretta@unipi.it (G.U.B.)

Table of contents

Figure S1. Spectral region of the ^{1}H NMR (600 MHz, $CDCl_3$, 25 °C) spectrum of compound **1** (10 mM) in equimolar CSA/**1**/DABCO mixture, including DNB resonances. Enantiomeric composition is 40% L- and 60% D-**1**.

Figure S2. Spectral region of ^{1}H NMR (600 MHz, $CDCl_3$, 25 °C) spectra involving amide nuclei of substrate **4** in 1:2 mixtures of CSA/**4** with: a) [CSA]=10 mM; b) [CSA]=0.7 mM.

Figure S3. Spectral region of ^{1}H NMR (600 MHz, $CDCl_3$, 25 °C) spectra involving amide nuclei of substrate **7** in 1:2 mixtures of CSA/**7** with: a) [CSA]=10 mM; b) [CSA]=5 mM; c) [CSA]=2.5 mM; d) [CSA]=1.25 mM.

Figure S4. Spectral region of ^{1}H NMR (600 MHz, $CDCl_3$, 25 °C) spectra involving chiral methine of substrate **9** in mixtures of CSA/**9** ([CSA]=5 mM) at the following CSA/substrate molar ratios: a) 1:2; b) 1:3; c) 1:4.

Figure S5. Spectral region of ^{1}H NMR (600 MHz, $CDCl_3$, 25 °C) spectra involving chiral methine of substrate **10** in mixtures of CSA/**10** ([CSA]=5 mM) at the following CSA/substrate molar ratios: a) 1:2; b) 1:3; c) 1:4.

Figure S6. Spectral region of ^{1}H NMR (600 MHz, $CDCl_3$, 25 °C) spectra involving chiral methine of substrate **11** in mixtures of CSA/**11** ([CSA]=5 mM) at the following CSA/substrate molar ratios: a) 1:2; b) 1:3; c) 1:4.

Figure S7. Spectral region of the ^{1}H NMR (600 MHz, $CDCl_3$, 25 °C) spectrum, including the chiral methine proton of substrate **9** in the presence of CSA (5 mM) at the stoichiometric ratio CSA/**9** 1:3.

Table S1. ^{1}H NMR (600 MHz, $CDCl_3$, 25 °C) nonequivalences ($\Delta\Delta\delta$, ppm) for selected resonances of racemic mixtures of substrates **15**-**20** in the presence of CSA **I** at different concentrations and molar ratios.

Figure S8. Spectral region of the ^{1}H NMR (600 MHz, $CDCl_3$, 25 °C) spectra, including the chiral methine of substrate **16** (a) and one of the two diastereotopic methylene protons of substrate **17** (b) in the presence of the CSA (5 mM) at CSA/substrate stoichiometric ratio of 1:2.

Figure S9. Spectral regions of ^{1}H NMR (600 MHz, $CDCl_3$, 25 °C) spectra, including the chiral methine (left) and methyl (right) protons of racemic mixtures of substrate **18** in the presence of the CSA (5 mM) at the following CSA/substrate stoichiometric ratios: a) 1:1; b) 1:2; c) 1:3; d) 1:4.

Table S2. ^{1}H NMR (600 MHz, $CDCl_3$, 25 °C) nonequivalences ($\Delta\Delta\delta$, ppm) for selected resonances of racemic mixtures of substrates **21**-**24** in the presence of CSA **I** at different concentrations and molar ratios.

Figure S10. Spectral region of the ^{1}H NMR (600 MHz, $CDCl_3$, 25 °C) spectrum including the chiral methine proton of substrate **22** in the presence of CSA (5 mM) at equimolar ratio.

Figure S11. ^{1}H NMR (600 MHz, $CDCl_3$, 25°C) spectrum of **9**/**I** mixture at the composition of 1:3 ([CSA] = 5 mM) (a) and 1D ROESY (600 MHz, $CDCl_3$, 25 °C, mixing time = 500 ms) spectra of the same mixture corresponding to: b) *ortho*-CH of substrate **9**; c) CH of substrate **9**; d) CH_3 of squaramide **I**. Protons involved in intermolecular ROE effects are indicated in the spectra.

Figure S12. ^{1}H NMR (600 MHz, 40 mM, DMSO-d_6 + 2 equiv of TFA, 25 °C) spectrum of compound **I**.

Figure S13. ^{13}C NMR (150 MHz, 40 mM, DMSO-d_6 + 2 equiv of TFA, 25 °C) spectrum of compound **I**. * Indicate resonances belonging to TFA.

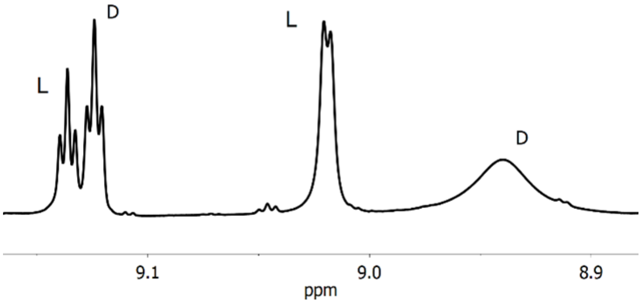

Figure S1. Spectral region of the ^{1}H NMR (600 MHz, $CDCl_3$, 25 °C) spectrum of compound **1** (10 mM) in equimolar CSA/**1**/DABCO mixture, including DNB resonances. Enantiomeric composition is 40% L- and 60% D-**1**.

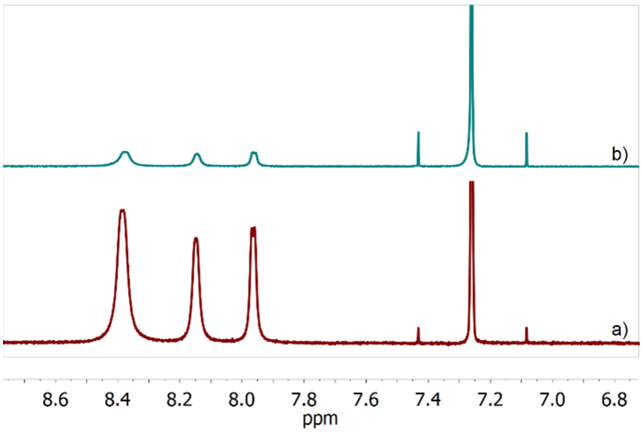

Figure S2. Spectral region of ^{1}H NMR (600 MHz, $CDCl_3$, 25 °C) spectra involving amide nuclei of substrate **4** in 1:2 mixtures of CSA/**4** with: a) [CSA]=10 mM; b) [CSA]=0.7 mM.

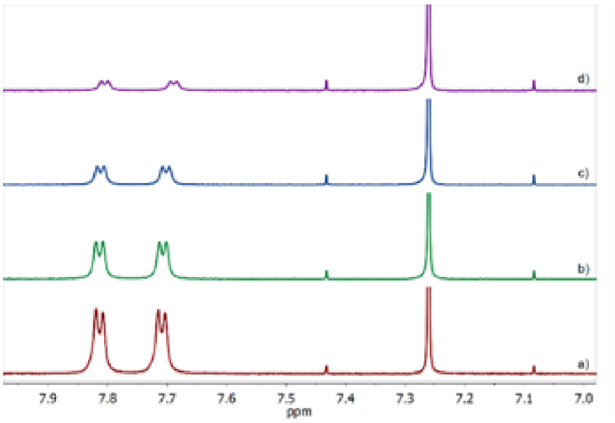

Figure S3. Spectral region of ^{1}H NMR (600 MHz, $CDCl_3$, 25 °C) spectra involving amide nuclei of substrate **7** in 1:2 mixtures of CSA/**7** with: a) [CSA]=10 mM; b) [CSA]=5 mM; c) [CSA]=2.5 mM; d) [CSA]=1.25 mM.

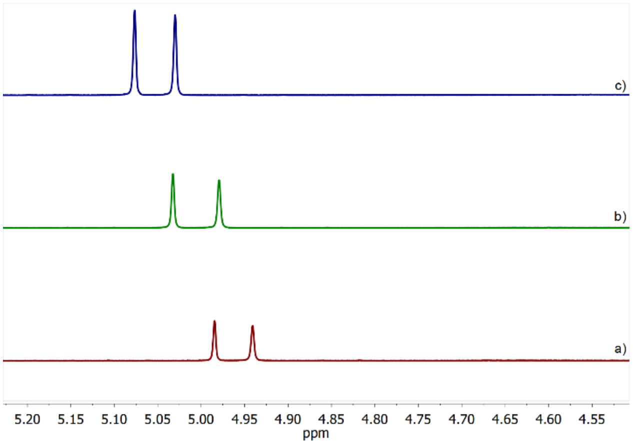

Figure S4. Spectral region of ^{1}H NMR (600 MHz, $CDCl_3$, 25 °C) spectra involving chiral methine of substrate **9** in mixtures of CSA/**9** ([CSA]=5 mM) at the following CSA/substrate molar ratios: a) 1:2; b) 1:3; c) 1:4.

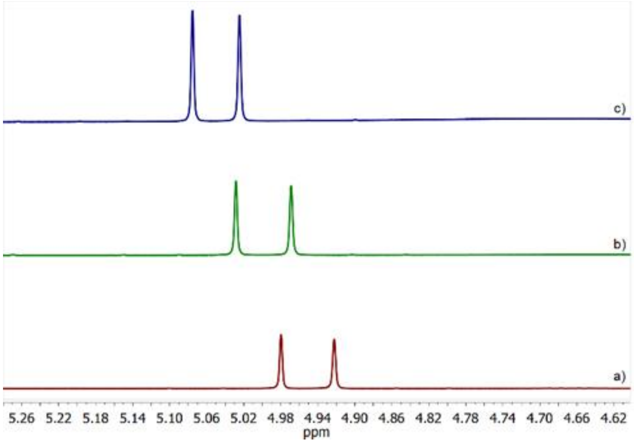

Figure S5. Spectral region of ^{1}H NMR (600 MHz, $CDCl_3$, 25 °C) spectra involving chiral methine of substrate **10** in mixtures of CSA/**10** ([CSA]=5 mM) at the following CSA/substrate molar ratios: a) 1:2; b) 1:3; c) 1:4.

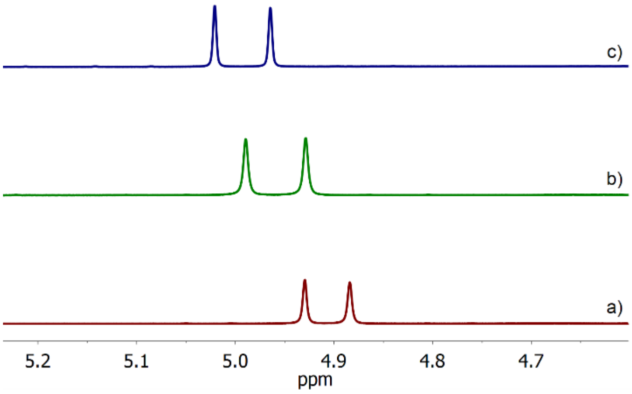

Figure S6. Spectral region of ^{1}H NMR (600 MHz, $CDCl_3$, 25 °C) spectra involving chiral methine of substrate **11** in mixtures of CSA/**11** ([CSA]=5 mM) at the following CSA/substrate molar ratios: a) 1:2; b) 1:3; c) 1:4.

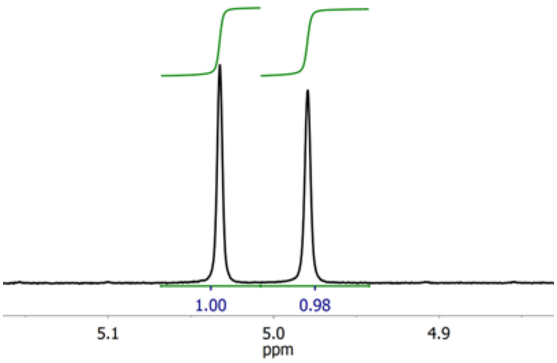

Figure S7. Spectral region of the ^{1}H NMR (600 MHz, $CDCl_3$, 25 °C) spectrum, including the chiral methine proton of substrate **9** in the presence of CSA (5 mM) at the stoichiometric ratio CSA/**9** 1:3.

Table S1. ^{1}H NMR (600 MHz, $CDCl_3$, 25 °C) nonequivalences ($\Delta\Delta\delta$, ppm) for selected resonances of racemic mixtures of substrates **15**-**20** in the presence of CSA **I** at different concentrations and molar ratios.

Substrate	CSA/Substrate	Concentrations	ΔΔδ (ppm)
**15**	1:1	CSA: 5 mM<br>Substrate: 5 mM	CH: 0.007<br>CH_3: 0.001
	1:2	CSA: 5 mM<br>Substrate: 10 mM	CH: 0.015<br>CH_3: 0.003
	1:3	CSA: 5 mM<br>Substrate: 15 mM	CH: 0.005<br>CH_3: 0.001
	1:4	CSA: 5 mM<br>Substrate: 20 mM	CH: 0.001
	1:2	CSA: 10 mM<br>Substrate: 20 mM	CH: 0.019<br>CH_3: 0.003
	1:2	CSA: 20 mM<br>Substrate: 40 mM	CH: 0.023<br>CH_3: 0.003
**16**	1:2	CSA: 5 mM<br>Substrate: 10 mM	CH: 0.130<br>Ar-CH*o*: 0.007; Ar-CH*m*: 0.005; Ar-CH*p*: 0.003
	1:3	CSA: 5 mM<br>Substrate: 15 mM	CH: 0.089<br>Ar-CH*m*: 0.002
	1:4	CSA: 5 mM<br>Substrate: 20 mM	CH: 0.070
**17**	1:1	CSA: 5 mM<br>Substrate: 5 mM	One of the diastereotopic protons of CH_2: 0.030<br>Ar-CH*o*: 0.026
	1:2	CSA: 5 mM<br>Substrate: 10 mM	CH: 0.013<br>One of the diastereotopic protons of CH_2: 0.076<br>Ar-CH*o*: 0.017<br>COOH: 0.548*<br>CH: 0.311*<br>CH_2: 0.421*
	1:3	CSA: 5 mM<br>Substrate: 15 mM	CH: 0.006<br>One of the diastereotopic protons of CH_2: 0.052
**19**	1:1	CSA: 5 mM<br>Substrate: 5 mM	CH: 0.007<br>CH_3 (propyl fragment): 0<br>CH_3 (isopropyl fragment): 0.003
	1:2	CSA: 5 mM<br>Substrate: 10 mM	CH: 0.012<br>CH_3 (propyl fragment): 0.003<br>CH_3 (isopropyl fragment): 0.002
	1:3	CSA: 5 mM<br>Substrate: 15 mM	CH: 0.012<br>CH_3 (propyl fragment): 0.003<br>CH_3 (isopropyl fragment): 0
	1:4	CSA: 5 mM<br>Substrate: 20 mM	CH: 0.009<br>CH_3 (propyl fragment): 0.003
**20**	1:2	CSA: 5 mM<br>Substrate: 10 mM	CH: 0.003<br>CH_3: 0.010
	1:3	CSA: 5 mM<br>Substrate: 15 mM	CH: 0.005<br>CH_3: 0.009
	1:4	CSA: 5 mM<br>Substrate: 20 mM	CH: 0.006<br>CH_3: 0.008
	1:5	CSA: 5 mM<br>Substrate: 25 mM	CH: 0.005<br>CH_3: 0.007
	1:4	CSA: 10 mM<br>Substrate: 40 mM	CH: 0.010<br>CH_3: 0.008

*^{13}C NMR (150 MHz, $CDCl_3$, 25 °C) nonequivalences ($\Delta\Delta\delta$, ppm).

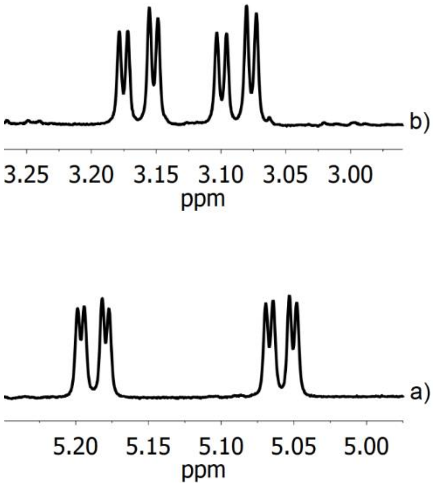

Figure S8. Spectral region of the ^{1}H NMR (600 MHz, $CDCl_3$, 25 °C) spectra, including the chiral methine of substrate **16** (a) and one of the two diastereotopic methylene protons of substrate **17** (b) in the presence of the CSA (5 mM) at CSA/substrate stoichiometric ratio of 1:2.

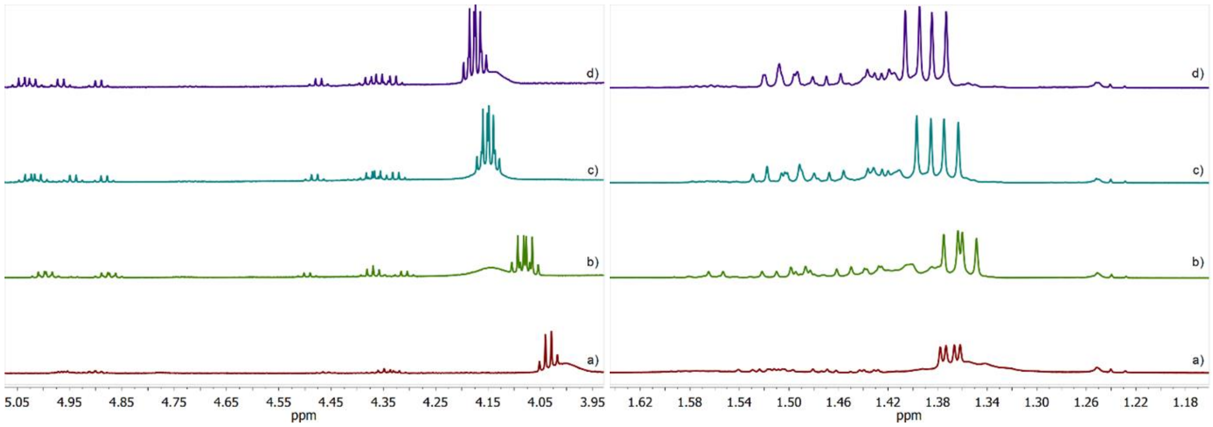

Figure S9. Spectral regions of ^{1}H NMR (600 MHz, $CDCl_3$, 25 °C) spectra, including the chiral methine (left) and methyl (right) protons of racemic mixtures of substrate **18** in the presence of the CSA (5 mM) at the following CSA/substrate stoichiometric ratios: a) 1:1; b) 1:2; c) 1:3; d) 1:4.

Table S2. ^{1}H NMR (600 MHz, $CDCl_3$, 25 °C) nonequivalences (ΔΔδ, ppm) for selected resonances of racemic mixtures of substrates **21**-**24** in the presence of CSA **I** at different concentrations and molar ratios.

Substrate	CSA/Substrate	Concentrations	ΔΔδ (ppm)
**21**	1:1	CSA: 5 mM<br>Substrate: 5 mM	CH_3 (main chain): 0.004<br>CH_3 (bound to CH): 0.007
	1:1	CSA: 10 mM<br>Substrate: 10 mM	CH_3 (main chain): 0.004<br>CH_3 (bound to CH): 0.008
	1:2	CSA: 5 mM<br>Substrate: 10 mM	CH_3 (main chain): 0.003<br>CH_3 (bound to CH): 0.004
	1:3	CSA: 5 mM<br>Substrate: 15 mM	CH_3 (main chain): 0.002<br>CH_3 (bound to CH): 0.003
**22**	1:1	CSA: 5 mM<br>Substrate: 5 mM	CH: 0.069<br>CH_3 (Ar): 0.012<br>CH_3 (Aliphatic chain): 0.012<br>CH(*meta* to CH_3 (Ar)): 0.026
	1:2	CSA: 5 mM<br>Substrate: 10 mM	CH: superimposed signals<br>CH_3 (Ar): 0.002<br>CH_3 (Aliphatic chain): 0.002<br>CH(*meta* to CH_3 (Ar)): 0.009
	1:3	CSA: 5 mM<br>Substrate: 15 mM	CH: superimposed signals<br>CH_3 (Aliphatic chain): 0.006<br>CH(*meta* to CH_3 (Ar)): 0.004
**23**	1:1	CSA: 5 mM<br>Substrate 5 mM	CH_3(a): 0.006<br>CH_3(b): 0.007
	1:2	CSA: 5 mM<br>Substrate 10 mM	CH_3(a): 0.007<br>CH_3(b): 0.003
	1:3	CSA: 5 mM<br>Substrate 15 mM	CH_3(a): 0.006
**24**	1:1	CSA: 5 mM<br>Substrate: 5 mM	CH: 0.025<br>Ar-CH*o*: 0.007; Ar-CH*m*: 0.003; Ar-CH*p*: 0.006
	1:2	CSA: 5 mM<br>Substrate: 10 mM	CH: 0.026<br>Ar-CH*o*: 0.007; Ar-CH*m*: 0.003; Ar-CH*p*: 0.004
	1:3	CSA: 5 mM<br>Substrate: 15 mM	CH: 0.025<br>Ar-CH*o*: 0.006; Ar-CH*m*: 0.003; Ar-CH*p*: 0.003
	1:4	CSA: 5 mM<br>Substrate: 20 mM	CH: 0.010<br>Ar-CH*o*: 0.005; Ar-CH*m*: 0.002; Ar-CH*p*: 0.003
	1:5	CSA: 5 mM<br>Substrate: 25 mM	CH: 0.009<br>Ar-CH*o*: 0.005; Ar-CH*m*: 0.002; Ar-CH*p*: 0.003
	1:6	CSA: 5 mM<br>Substrate: 30 mM	CH: 0.006

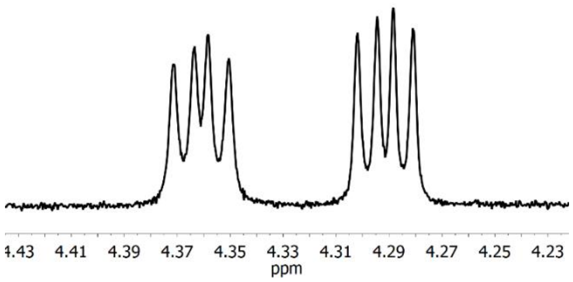

Figure S10. Spectral region of the ^{1}H NMR (600 MHz, $CDCl_3$, 25 °C) spectrum including the chiral methine proton of substrate **22** in the presence of CSA (5 mM) at equimolar ratio.

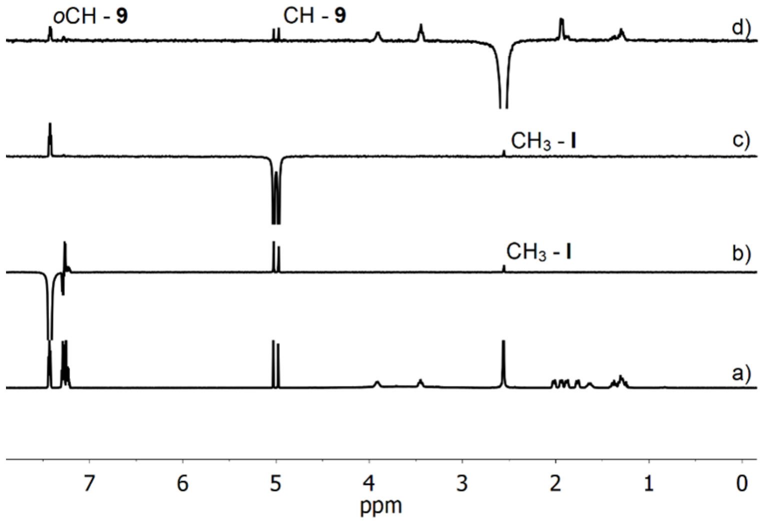

Figure S11. ^{1}H NMR (600 MHz, $CDCl_3$, 25°C) spectrum of **9**/**I** mixture at the composition of 1:3 ([CSA] = 5 mM) (a) and 1D ROESY (600 MHz, $CDCl_3$, 25 °C, mixing time = 500 ms) spectra of the same mixture corresponding to: b) *ortho*-CH of substrate **9**; c) CH of substrate **9**; d) CH_3 of squaramide **I**. Protons involved in intermolecular ROE effects are indicated in the spectra.

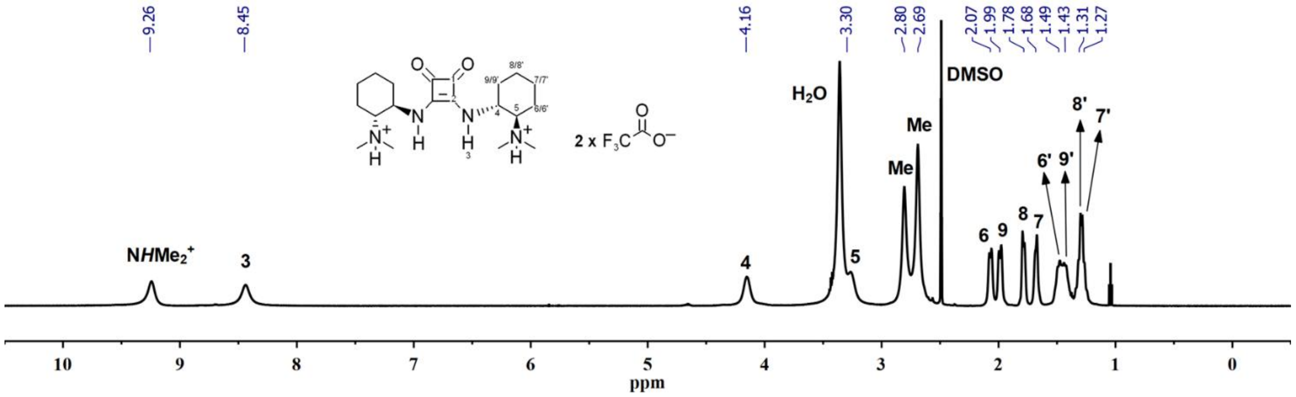

Figure S12. ^{1}H NMR (600 MHz, 40 mM, DMSO-d_6 + 2 equiv of TFA, 25 °C) spectrum of compound **I**. The apex indicates the axial proton.

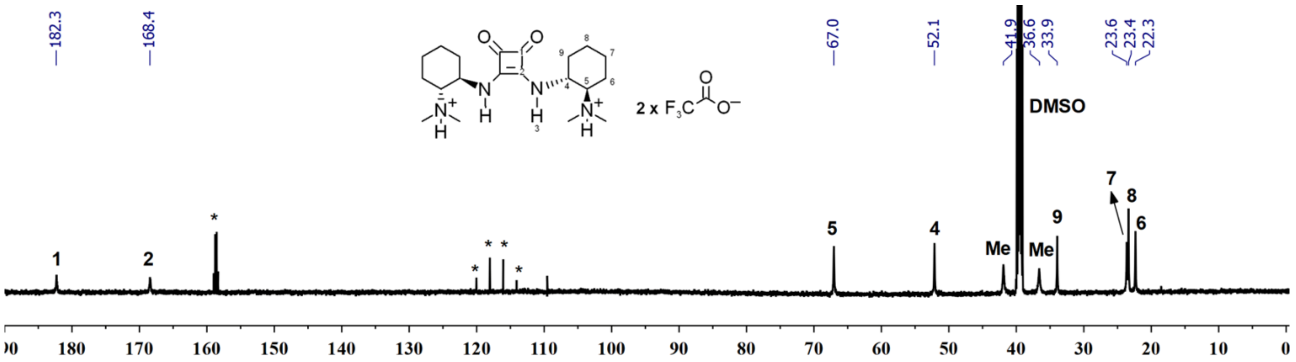

Figure S13. ^{13}C NMR (150 MHz, 40 mM, DMSO-d_6 + 2 equiv of TFA, 25 °C) spectrum of compound **I**. * Indicate resonances belonging to TFA.
